# Anesthetic and Airway Management in a Case of Surgical Excision of Recurrent Buccal Hemangioma

**DOI:** 10.7759/cureus.39951

**Published:** 2023-06-04

**Authors:** Shrilekh Mankhair, Jui A Jadhav, Vivek Chakole, Amol Singam, Neeta Verma

**Affiliations:** 1 Department of Anesthesiology, Datta Meghe Institute of Medical Sciences (Deemed to be University), Wardha, IND; 2 Department of Anesthesiology, Jawaharlal Nehru Medical College, Datta Meghe Institute of Medical Sciences (Deemed to be University), Wardha, IND; 3 Department of Anesthesia, Jawaharlal Nehru Medical College, Datta Meghe Institute of Medical Sciences (Deemed to be University), Wardha, IND

**Keywords:** pulmonary aspiration, ventilation, difficult airway, intubation, bleeding, buccal hemangioma

## Abstract

Difficult airway is one of the challenges trained anesthesiologists face in their life.

Induction of general anesthesia in a patient with a compromised airway has always caused a dilemma for anesthesiologists. Challenges were more in this case of buccal hemangioma as its bleeding tendency makes it a challenging job. Hemangioma is a benign vascular anomaly characterized by rapid endothelial cell proliferation. It appears within the first eight weeks of life, rapidly proliferates between the ages of six and 12 months, and progressively involutes between the ages of nine and 12 years. Hemangiomas are more common in women, with a male-to-female ratio of 1:3 to 1:5. By the age of nine years, over 80%-90% of hemangiomas have completely involuted. The remaining 10%-20% involute incompletely, necessitating post-adolescent ablative treatment or alternative management options. Hemangiomas in the head and neck region account for 50%-60% of all hemangiomas. Intra-orally, the lips, buccal mucosa, and tongue are the most prevalent sites of involvement. Here, we report a case of recurrent left-sided buccal hemangioma in a 20-year-old female patient. Treatment options available to manage hemangioma include cryotherapy, laser ablation therapy, radiotherapy, sclerotherapy, and selective embolization. After prophylactic embolization of feeder vessels, surgically excising the lesion is the modality of choice. So from a general anesthesia management point of view, buccal hemangioma poses multiple challenges including difficulty in mask ventilation, difficulty in intubation, bleeding, and pulmonary aspiration.

## Introduction

Difficult airway is one of the most challenging situations for even a trained anesthesiologist. Induction of general anesthesia in a patient with a compromised airway due to buccal hemangioma has always caused a dilemma for the anesthesiologist. Hemangioma is a benign vascular anomaly caused by the fast proliferation of endothelial cells [1]. It is not visible at birth but appears within the first month of life. Hemangiomas are most typically found in the head and neck [2]. Lips, tongue, buccal mucosa, and palate are common intraoral locations [3]. Hemangiomas come in a variety of sizes, shapes, and morphologies. When the superficial dermis is affected, the skin becomes elevated, hard, and crimson in color. The overlying skin may be somewhat elevated, heated, and have a bluish tinge if the hemangioma is extension-wise limited to the deeper dermis and/or subcutaneous tissue or muscle. With a superficial elevated component atop a deeper tumor, any of these tissues could be involved [4]. Hemangiomas are brittle growths that bleed abundantly when touched, so care should be taken to avoid manipulation of hemangioma and also the risk of aspiration of blood must be kept in mind during general anesthesia induction. The size, location, and nature of hemangiomas many times make even mask ventilation difficult, making the anesthesiologist either go for rapid sequence induction or spontaneous induction of general anesthesia. As a result, the anesthesiologist must determine the best course of action for inducing general anesthesia. This research paper highlights a case of management of the airway in buccal hemangioma that was detected and treated surgically based on clinical, radiographic, and histological findings. The airway management was decided based on the anticipation of events, risk assessment, and available resources.

## Case presentation

**Figure 1:**
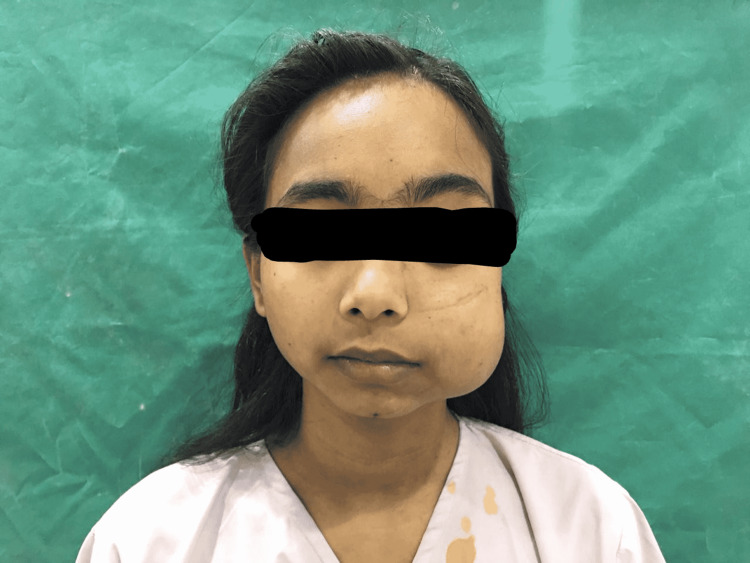
Buccal hemangioma

**Figure 2:**
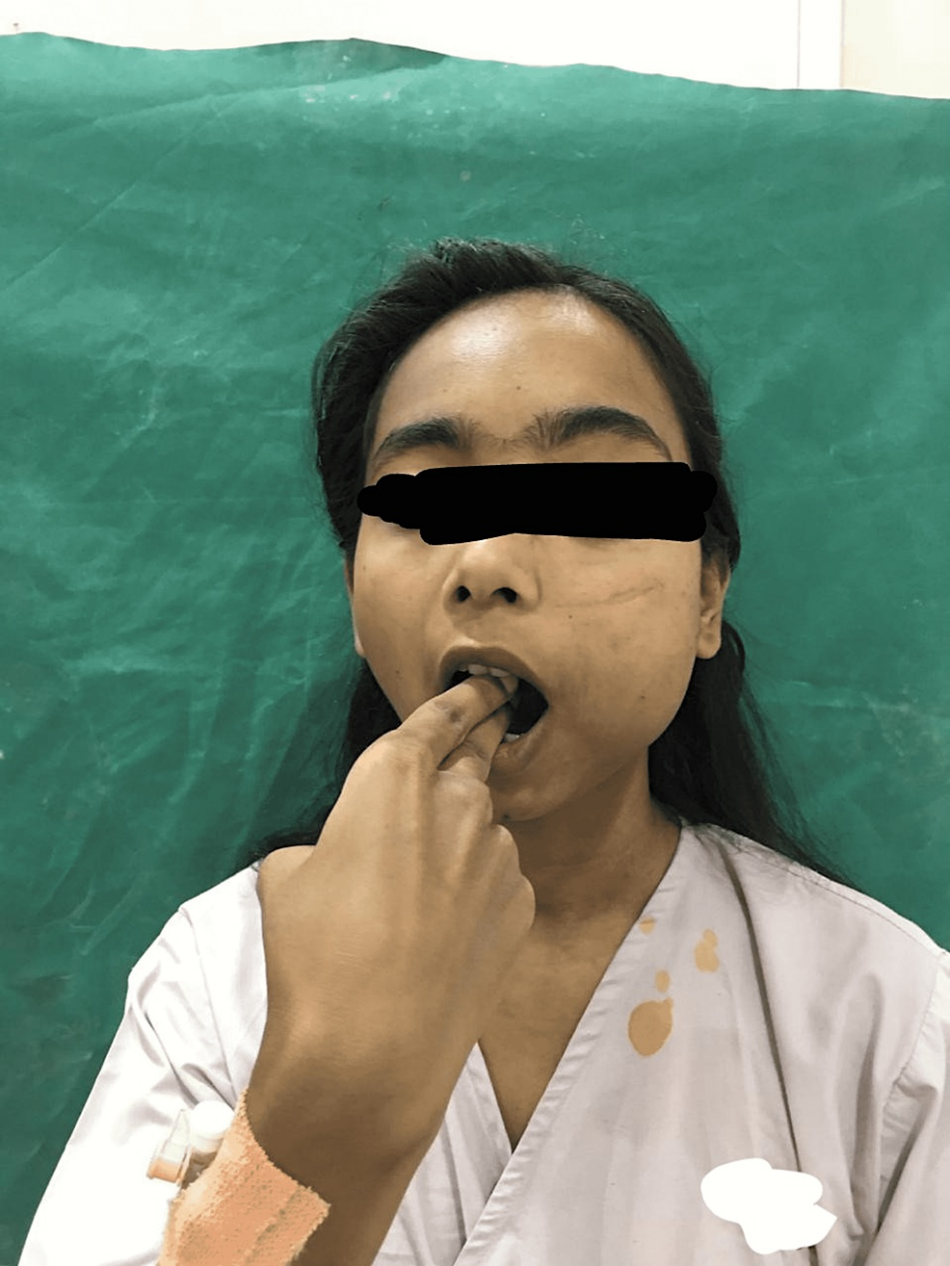
Mouth opening

**Figure 3:**
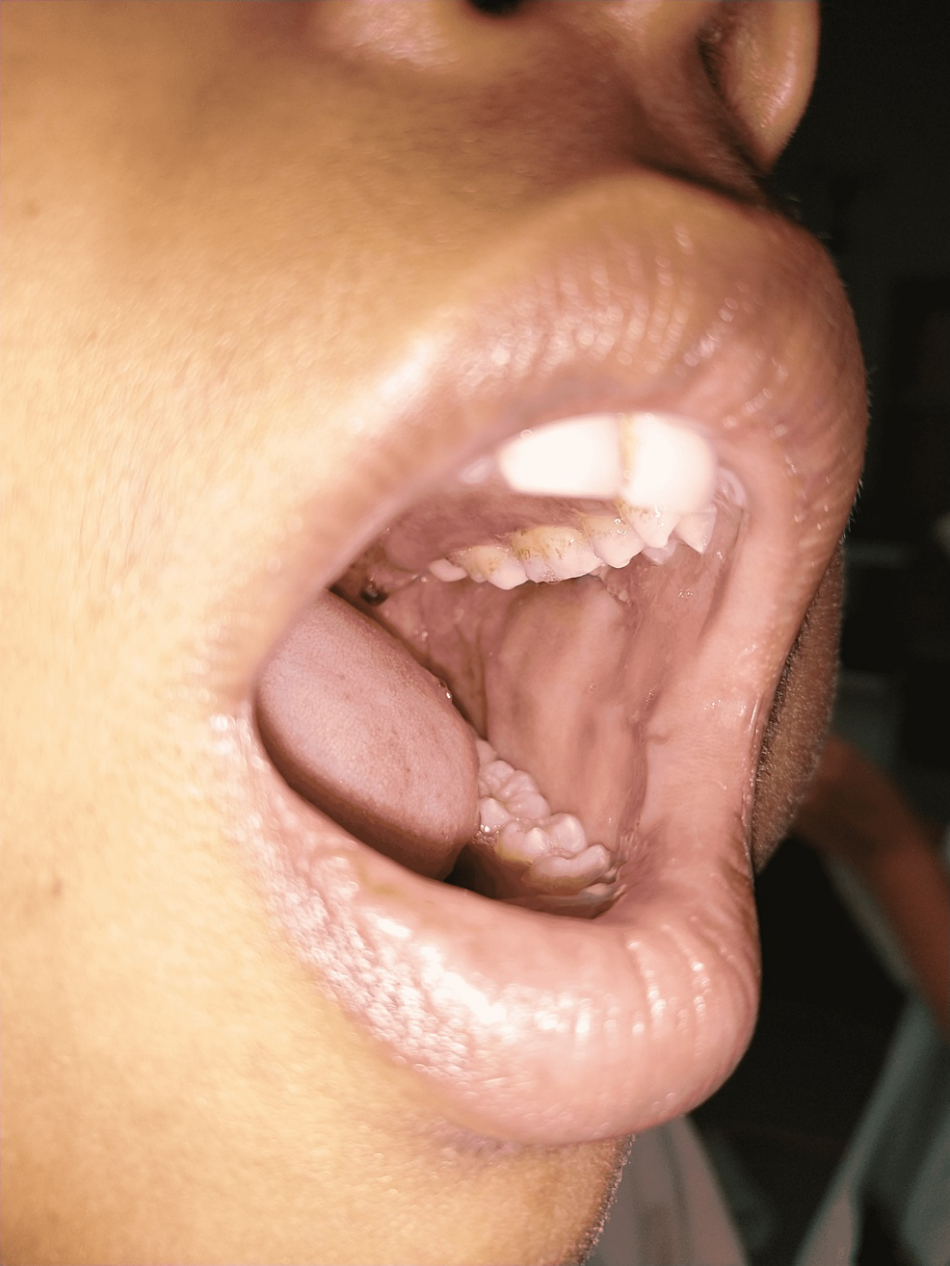
Intraoral view of hemangioma

A 20-year-old female patient reported to our hospital with a chief complaint of intraoral swelling on the left-side buccal mucosa (Figure 1). The patient became aware of the swelling 15 years back and the swelling was found to have sudden onset and non-progressive in nature. The swelling was associated with intermittent pain and the pain increased on intake of spicy food. The patient was having a mouth opening enough to accommodate barely two fingers (Figure 2). With this much mouth opening intraoral examination was done where it was found to be a solitary, well-differentiated, nodular lesion with a normal mucosal covering on the left buccal mucosa. Though the surrounding surface was regular, the lesions overlying the surface were irregular (Figure 3). On palpation, the lesion was compressible and soft, painful on compression. The lesion blanched on pressure. Depending on the clinical scenario encountered, the provisional diagnosis of hemangioma was made. The patient was operated on for similar hemangioma at the age of one and two years, respectively, and was also operated on for orbital swelling on the left lower eyelid at the age of 10 years. After orbital mass excision surgery, the patient was having a complaint of bleeding from the left nostril, which was 1-2 episodes every month. Also, the patient complains of a running nose and sneezing on exposure to the cold.

Routine blood investigations were normal. Intravenous DSA (digital subtraction angiography) was performed for this highly vascular mass in the left cheek, which was suggestive of hemangioma. So, prophylactic tumor embolization of feeder from branches of the left-side external carotid artery was done as a prophylactic tumor embolization before performing excision surgery. 

**Figure 4:**
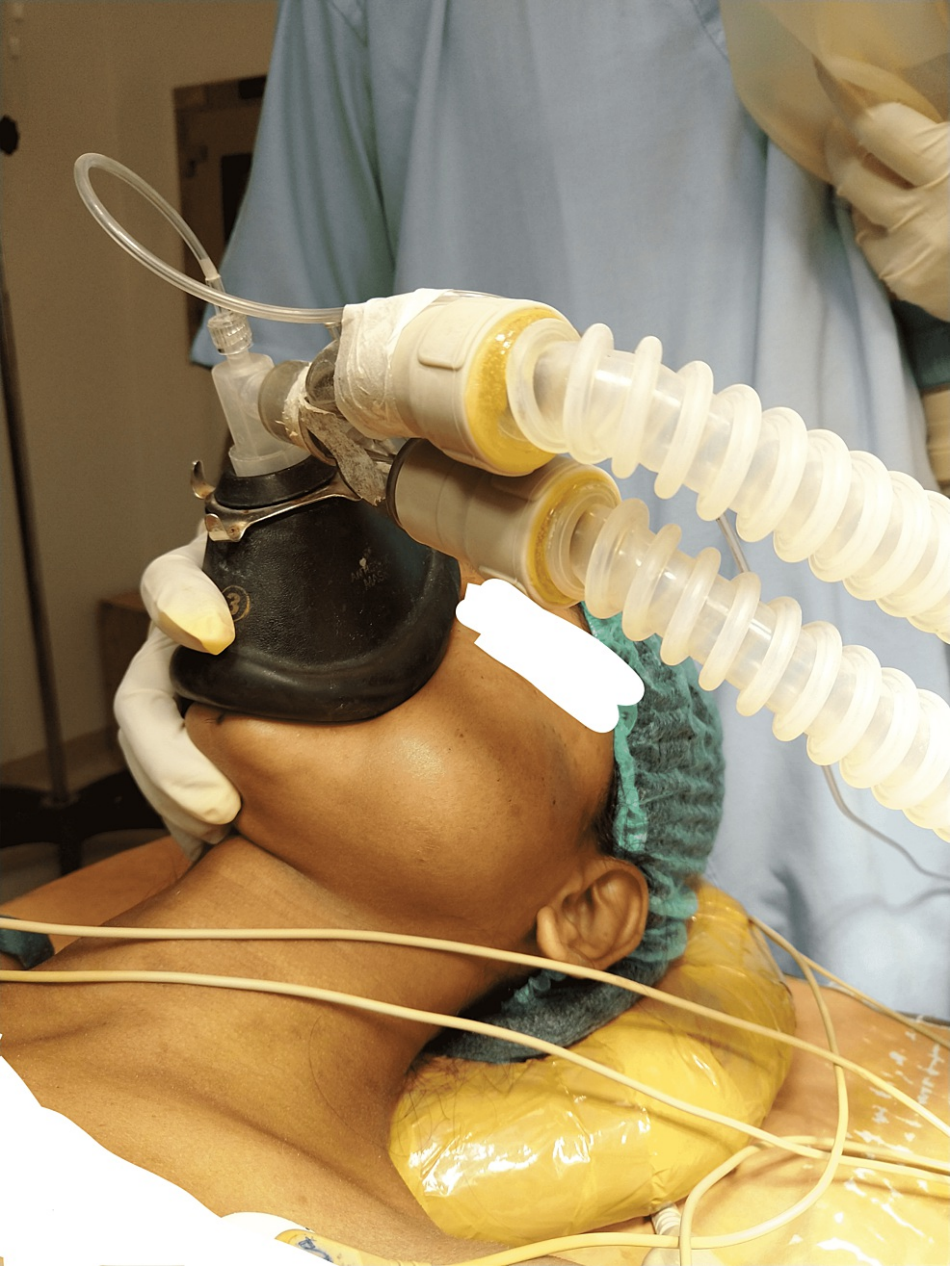
Mask ventilation

**Figure 5:**
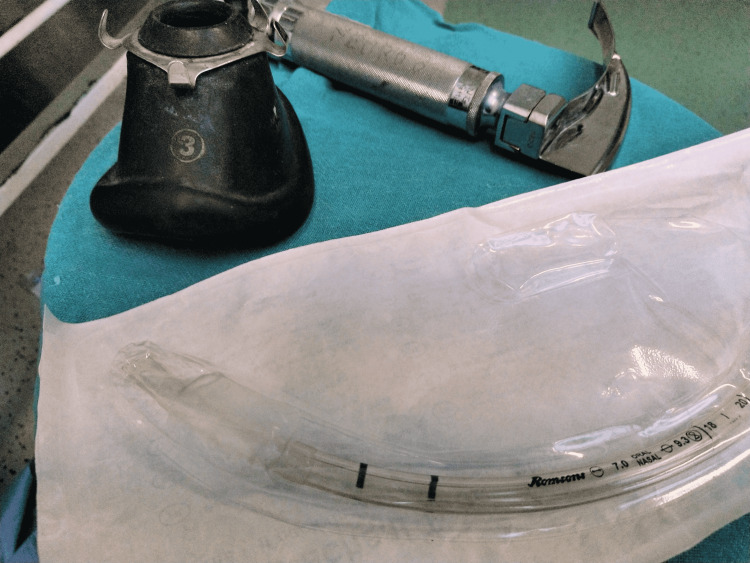
Macintosh blade no. 3 with 7 mm internal diameter polyvinyl chloride endotracheal tube, no. 3 anatomical mask

**Figure 6:**
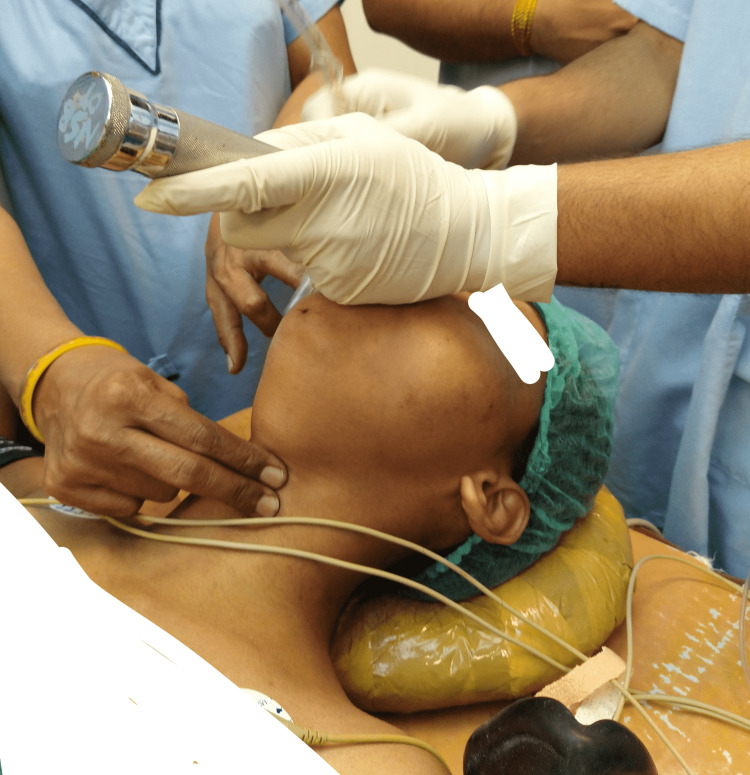
Endotracheal intubation with external cricoid manipulation

Anesthetic concerns that were faced before and during surgery were difficult mask ventilation and difficult intubation, risk of bleeding from hemangioma, and risk of aspiration of blood. The LEMON score was used to assess the airway during pre-anesthetic check-up. The LEMON score stands for Look, Evaluate, Mallampati grade, Obstruction, and Neck mobility. All these components of the LEMON score help to assess the airway during pre-anesthetic check-up. It showed the anatomical feature of facial/oral swelling; the oral opening was shown to accommodate two fingers barely, and Mallampati grading was grade II suggesting a potentially difficult airway (Figures 1, 2). A size of 3 cm × 4 cm and a painful buccal mass posed a challenge for mask holding and ventilation. To overcome this challenge, no. 3 anatomical mask was held with the right hand from the right side without any pressure on soft tissue, making sure that it does not put pressure on the left cheek mass (Figure 4). To overcome the fear of bleeding, prophylactic preoperative embolization of feeder vessels from the left external carotid artery was done. Hemangiomas, being friable vascular tumors, are prone to bleed profusely leading to aspiration of blood during laryngoscopy manipulations, making laryngoscopy challenging during intubation. Use of awake nasotracheal fiber-optic intubation would have been safest to avoid laryngoscopy intubation-related risk of bleeding from hemangioma but as the patient was having a complaint of bleeding from nostrils and also there was a presence of deviated nasal septum on the left side, fiber-optic intubation was avoided. The video laryngoscope was not available. The surgeon was asked for a back-up emergency tracheotomy in the event of failed intubation. This challenge was tackled by doing rapid sequence intubation with an injection of succinylcholine by a trained anesthesiologist in a single attempt with a laryngoscope attached by no. 3 Macintosh blade, which was introduced from the right corner of the mouth. While attempting intubation, the laryngoscopy was done with the least possible manipulations to avoid the event of bleeding from the hemangioma. With external laryngeal manipulations a 7 mm internal diameter endotracheal tube was inserted till the vocal cord marker passed the vocal cords, and after achieving that depth the endotracheal tube was secured at 20 cm marking at the level of the right upper incisor (Figures 5, 6). To prevent aspiration, a throat gauge pack was inserted on both sides of the endotracheal tube. During rapid sequence induction intubation, intravenous drugs were used as follows: injection of glycopyrrolate 0.2 mg, injection of fentanyl 100 microgram, injection of propofol 100 mg, and injection of succinylcholine 100 mg. After intubation and on return of spontaneous ventilation, non-depolarizing neuromuscular blocking agent injection vecuronium was given at the dosage of 6 mg intravenously. For maintenance of anesthesia, sevoflurane was used as as inhalation agent with fresh gas flow containing oxygen and air in a ratio of 50:50.

Surgical excision took 1 hour 30 minutes, and with proper assessment of the patient and after giving reversal agent neostigmine 2.5 mg and glycopyrrolate 0.5 mg and injection hydrocortisone 100 mg, the patient was reversed and thorough suctioning was done. Injection paracetamol was given at the dose of 15 mg per kilogram weight toward the end of surgery for analgesia. After visible adequate spontaneous respiration, spontaneous eye-opening, and when the patient started following verbal commands, she was extubated without any issues. The patient was then shifted to the postoperative care unit.

With proper assessment and anticipating the limitations, the case was managed with routine general anesthesia induction with routine laryngoscopy without taking the help of any advanced airway instruments. The difficult airway cart was kept ready with an intersurgical I-gel supraglottic airway device of sizes 2.5 and 3. The surgical team was asked to be ready for emergency surgical tracheostomy in the event of failure of intubation. All the above procedures and risks were explained to the patient and her guardians and proper written consent was taken for the same.

## Discussion

Hemangioma is a term that has been used to describe a range of vascular malformations that occur during infancy and youth. Although just a few cases have been found to be congenital, most hemangiomas are not visible at birth and develop during the first eight weeks of life. Buccal mucosa (45.2%) is the most prevalent site for intraoral hemangiomas, followed by the tongue (35.5%), lip (9.7%), gingiva (6.5%), and palate (3.2%) [5]. Clinically and histopathologically, hemangiomas might seem like lesions such as epulis granulomatosa, pyogenic granuloma, chronic inflammatory gingival hyperplasia (epulis), squamous cell carcinoma, and peripheral giant cell granuloma. Hemangiomas do not require treatment when they are young since they spontaneously shrink. Only 10%-20% require treatment which is based on various factors such as the age of the patient, clinical features, and the anatomic considerations [6]. The most common treatment of choice for hemangioma is surgical excision of the lesion with or without ligation of vessels and embolization. This case is of a 20-year-old female who is a known case of recurrent hemangioma of the left cheek for 15 years. The patient was operated on in the past for a similar hemangioma at the age of one year and two years. The patient underwent surgery for the orbital mass of the lower eyelid on the left side 10 years back. Since then, according to the patient she is having episodes of bleeding from the left nostril as mentioned earlier. Also, the patient complains of a running nose and sneezing on exposure to the cold. Looking at the complaint of recurrence of the lesion, difficulty in mastication, and also pain, it was decided to excise the lesion surgically. The intravenous DSA was done, which was suggestive of left cheek hemangioma with feeders from the branches of the left external carotid artery. So the prophylactic hemangioma embolization was planned 48 hours before surgical excision. The preoperative prophylactic hemangioma embolization was done 48 hours prior, that is two days prior to the surgical excision hemangioma. The procedure was done under all aseptic precautions via right femoral artery access. Post-embolization report showed a complete embolization of vascular supply. Right lower limb immobilization suggested post-embolization procedure. The procedure was uneventful.

Intraoral lesions including hemangiomas pose multiple challenges for airway management like difficult mask ventilation, reduced mouth opening, obstruction to visualization during intubation, risk of bleeding leading to blood loss, and chances of aspiration (Figure 3) [7]. To overcome all these challenges and perform airway management, fiber-optic nasotracheal intubation may be preferred and is a better option but it is a matter of expertise and availability. Also in our case, the patient was having complaints of monthly episodic bleeding from nostrils and deviated nasal septum in the left side, so routine oral intubation was the option. A video laryngoscope can also be used for intubation, thereby increasing the success rate of intubation and reducing the chances of damage to the friable hemangioma. video laryngoscope is a matter of expertise and availability. In this case, the working video laryngoscope was not available. The supraglottic airway devices can also be an alternative but the risk of device dislodgment followed by aspiration cannot be taken care of with supraglottic airway devices. An elective surgical airway can be secured with tracheostomy but then it increases the patient's hospital stay and postoperative tracheostomy care. So after anticipating all challenges and problems, the patient was intubated with rapid sequence induction intubation with external laryngeal manipulations using Macintosh blade no. 3 and 7 mm endotracheal tube was inserted and secured at 20 cm marking at the level of the right upper incisor. An oral suction catheter of size 14 was kept ready with the suctioning machine on throughout the procedure of intubation. A surgeon was asked for a standby emergency tracheostomy in the event of failed intubation [8]. Time and the duration of the surgery also decide whether an extubation trial shall be given or not. As here surgery was performed in 1 hour and 30 minutes and much edema was not anticipated, the patient was successfully extubated.

Surgical excision took 1 hour 30 minutes, and with proper assessment of the patient and after giving reversal agent neostigmine 2.5 mg plus glycopyrrolate 0.5 mg and injection hydrocortisone 100 mg, the patient was reversed and extubated without any issues and shifted to the postoperative care unit.

## Conclusions

With proper assessment and evaluation of the difficulties in airway management of such recurrent buccal hemangioma, cases can be managed without using advanced airway instruments.
